# What to do about portion sizes? Roundtable discussion at the forefronts in portion size conference

**DOI:** 10.1038/ijo.2014.87

**Published:** 2014-07-25

**Authors:** H A Raynor

**Affiliations:** 1 Department of Nutrition, University of Tennessee, Knoxville, TN, USA

## Abstract

As portion size has increased in the United States, greater concern has arisen regarding the positive relationship between portion size and consumption, and how this relationship may negatively impact weight management. This article is a summary of a roundtable discussion from the Forefronts in Portion Size Conference held in Philadelphia, PA, USA in May 2013. The roundtable included four discussants, five speakers who had presented research on various components of portion size at the conference, two organizers and the moderator. Topics discussed included methods to reduce portion size that can assist with reducing energy intake, societal norms about portion size, values associated with portion size and methods to promote smaller portion sizes. Areas needing additional research were also identified.

## Introduction

The aim of this paper is to summarize the roundtable discussion at the conclusion of the five presentations at the Forefronts in Portion Size Conference that was held in Philadelphia, PA, USA on 3 May 2013. This conference provided a forum for discussion dedicated exclusively to research regarding the impact of portion size on consumption; the effect of learning on decisions made about portion sizes; the influence of environmental interventions that include marketing and behavioral economic paradigms on choices concerning portion size and consumption; and the role of labeling in informing decisions about portion size. The presentations included: (1) What is the role of portion size in weight management? (presenter: Barbara Rolls, PhD); (2) How much is enough? A role for ‘expected satiety' in decisions about portion size (presenter: Jeffery Brunstrom, PhD); (3) Opportunities and barriers for smaller portions: lessons from marketing and behavioral economics (presenter: Jason Riis, PhD); (4) Small, medium, large or supersize? The development and evaluation of interventions targeted at portion size (presenter: Willemijn Vermeer, PhD); and (5) Can labeling help consumers make healthier portion-size decisions? (presenter: Christina Roberto, PhD). Discussants involved in the roundtable were Johanna Dwyer, PhD; Tanja Kral, PhD; Sylvia Rowe; and Thomas Wadden, PhD. The roundtable discussion also included the speakers, coordinators of the conference (Jennifer Orlet Fisher, PhD, and Gary Foster, PhD) and the moderator, Hollie Raynor, PhD.

The roundtable discussion started on the premise of several concepts that had been provided in the presentations. The first premise was that in the United States, portion sizes of commonly available foods have increased during the past 30 years, which has coincided with the timeframe in which the prevalence of obesity has increased.^1,2^ Basic behavioral research has found that consumption increases when foods are served in larger portions, and that compensation to the initial increased intake, via consuming smaller amounts from other foods within a meal or consuming an overall reduced amount over time, does not occur.^3,4^ This indicates that larger portion sizes can contribute to excessive energy intake, which could be problematic for weight management. Additionally, learning from previous experiences with portion sizes regarding satiation capabilities can influence decisions about portion sizes prior to actual consumption.^5,6^ Decisions regarding size of the portion to consume within an eating bout can occur using either reflective (slow) or automatic (fast) thinking and interventions designed to influence decisions regarding portion size can target both areas.^7,8^ Interventions that target reflective thinking would require strategies to increase awareness regarding portion size, whereas those that target automatic thinking could make the smaller-portion decision the ‘default' choice. One challenge for interventions regarding portion size is the relationship between perceived value and quantity, such that the ability to purchase larger portions of food at ‘lower' prices (value priced sizing) is highly regarded.^9,10^ Moreover, it does not appear that the relationship between portion sizes and health is readily within the public's awareness. Finally, current nutrition labels (front of packaging, Nutrition Facts panel, or menu labeling) do not appear to be consistently viewed, understood, or used to make decisions about portion size by consumers.^11,12^

## Methods for downsizing portion size to impact energy intake

The 2010 Dietary Guidelines encourage individuals to, ‘prepare, serve, and consume smaller portions of foods and beverages,' and to ‘avoid oversized portions'.^13^ This guidance acknowledges that the normative portion size that many Americans use is too large, and thus the portion size that should be used for consumption needs to be smaller. However, no description is given regarding what the portion size should be, or how much the normative portion size should be reduced. Thus, there is a lack of clarity about what constitutes a ‘smaller' or ‘ideal' smaller portion. If individuals do not regularly measure foods to assist with downsizing their portion (that is, know that they are consuming a smaller portion than what they have consumed in the past, or recognizing that their portion is three times the size of a standard serving), methods other than measuring food are required in assisting with eating smaller portions.

It is clear that larger portion sizes increase consumption outside of awareness, and that the increased consumption does not alter feelings of hunger, satiation or satiety.^3,4^ An important scientific and clinical question is how much can a portion be decreased, such that there is a meaningful reduction in intake, but that hunger, satiation and satiety are not altered? Ideally the maximum reduction in portion size that decreases consumption outside of awareness (that is, where there are no differences in hunger, satiation and satiety) can be identified. The amount of reduction could then serve as a guide for defining a smaller portion. Other strategies could be combined with this ‘downsized' portion to assist with reduced awareness of the reduction in the size of the portion. Packaging could potentially assist with creating the illusion that no change in portion size has occurred. For example, packaging that is long and narrow, rather than short and wide, shows promise in assisting with smaller portions not being noticed.^14^ These options could target automatic thinking and serve as the ‘default' choice. Implementation of this downsized portion by industry could provide the public with a ‘nudge' towards consuming smaller portions.

Additionally, different guidelines regarding portion size may be needed for different types of food. Energy density also influences energy consumption outside of unawareness. Foods lower in energy density allow for the same amount of food to be consumed with a lower energy intake, as compared with foods higher in energy density. Therefore, although downsizing the portion of foods high in energy density may be important for reducing energy intake, a reduction in portion size for foods low in energy density would not influence energy intake as much and may not be necessary. Furthermore, actually increasing the portion size of low-energy-dense foods may assist with reducing the portion size of high-energy-dense foods when both foods are consumed within the same meal or snack. Redistributing the amount of food consumed in an eating bout so that a greater portion of low-energy-dense foods are consumed as compared with high-energy-dense foods (that is, substitution) could allow the overall amount of food consumed in the eating bout to stay constant, but reduce energy intake. The graphic of ChooseMyPlate and the consumer message to ‘make half of your plate fruits and vegetables' highlights the concept of consuming a large portion of low-energy-dense foods (most fruits and vegetables are low in energy density).^15^ Having different portion sizes for different types of food to promote substitution of low- for high-energy-dense foods could be accomplished using both reflective (that is, specific guidelines provided to assist which choices made during meals and snacks, prompting at the point of purchase) and automatic (that is, purchased food automatically prepared in this manner and modeling recommendations regarding portion size of low- and high-energy-dense foods) thinking.

## Societal norms for portion size

As portions sizes have increased in the United States over these past few decades, the norms regarding the size of an ‘appropriate' portion most likely has increased. This may make it more challenging for the public to follow recommendations that emphasize standard serving sizes, as the difference in size between the standard serving and the normative portion has increased. Potentially, norms regarding portions size need to be altered, so that portions smaller than what are currently consumed are considered to be more optimal. Several strategies could be used to alter portion-size norms. One strategy would be increased exposure to smaller portions. The size of the reduction may not need to be so great as to make a single portion equal to the size of a standard serving, but a large enough reduction so that the amount is smaller than current portion sizes. The size of the reduction if too great may be perceived as being unrealistic, which would not assist with changing norms. The amount of exposure to smaller portions needed to change portion-size norms is not clear. For example, what is the ratio of smaller to larger portion sizes needed to alter perception of portion size (that is, do 75% of the items available need to be of a smaller portion size to have an impact?)? How many different types of foods in smaller portions does one need to be exposed (that is, does exposure to smaller portions generalize well to foods with similar characteristics)? Finally, what is the length of time of exposure to smaller portions required to change norms?

In addition to exposure, social marketing could be another strategy used to assist with developing positive connotations about smaller portions, helping with making the smaller portion acceptable and normative. Campaigns using celebrities, athletes and other societal positive role models could be developed in which models are shown consuming smaller portions and promoting positive aspects of eating smaller portions. Increasing overall exposure to smaller portions and developing positive associations about consuming smaller portions may both be needed to alter portion-size norms. Decreasing the norm about the size of an ‘appropriate portion' could make preparation and consumption of a smaller portion an easier behavior to accomplish.

## Portion size and values

Portion size and food cost have been strongly linked for consumers. This relationship has emphasized value-priced sizing, which promotes purchasing larger, rather than smaller, sized options. To combat the message of ‘get more for your dollar,' positive values related to downsized portions need to be developed and promoted. Although the Dietary Guidelines recommend smaller portions, the relationship between portion size, intake, weight status and health may be vague and unclear to many consumers. Thus, messages framed around the relationships between consuming smaller portions and enhanced weight management, which can lead to improved health, reduced health care costs and enhanced quality of life need to be developed. Social marketing could assist with the promotion of these messages, which could positively reframe the value of a smaller portion.

## Promotion of smaller portion sizes

To change societal norms about ‘appropriate' portion size and values about consuming smaller portions, comprehensive national campaigns and/or policy changes need to occur. Very explicit changes in portion sizes to enhance exposure to smaller portions would also require large-scale industry changes. However, there are concerns from consumers about the manner in which changes may originate owing to feelings of ‘paternalism', in which the government is making decisions about food portions that limits the ability of consumers to make decisions regarding their own behaviors around portion size and overall consumption. To address this concern and potentially reduce the perception of paternalism, others may need to take the lead on these issues. Industry could be a leader, if these types of promotions are not perceived as negatively affecting profit. Healthcare could also contribute to comprehensive reform in these areas, as ultimately consuming smaller portions is believed to be important for improving the nation's health.

## Futures areas of needed research

Although it is evident that the large portion sizes available in the US are contributing to excessive intake and may, therefore, be a factor in the increased prevalence of overweight and obesity, many areas about portion size are not clear. The roundtable discussion identified some specific areas in which research is needed:

1. What is the degree in reduction of portion size that needs to occur that will produce a meaningful reduction in energy intake that can assist with achievement of a healthy weight status?
2. What is the degree of reduction in portion size that can occur that will produce a meaningful reduction in energy intake, but not be perceived, both visually and in terms of appetite regulation sensations, as a reduction in portion size?
3. What is the optimal portion size for low-energy-dense foods and high-energy-dense foods to assist with substitution of low-energy-density for high-energy-density foods?
4. What components of exposure to a smaller portion sizes (that is, number of different types of foods, ratio of smaller to larger portion sizes) can assist with changing choices about portion sizes (that is, choose the smaller portion) and norms about portion sizes?
5. Can social marketing assist with changing portion size norms, reducing the connection between value and portion size and increasing the association between health and portion size?
6. What produces the best outcomes, smaller, covert, less noticeable changes (‘nudges') or larger, overt changes?
7. As multiple levels of intervention (that is, targeting reflective and automatic thinking which could involve individual, industry and policy changes) may be required, determining the best combination to assist with reducing portion sizes consumed, and address consumers concerns about paternalism, is important.

## Conclusion

This conference highlighted that research conducted on the topic of portion size is in its infancy. Although it is clear that portion sizes have increased and larger portion size enhances consumption, it is not known what degree of portion-size reduction is needed to meaningfully decrease intake, what amount of reduction is feasible to implement or what is the best method(s) to reduce portion size for long-term outcomes. Research that can address these areas is greatly needed.

## Notes

Dr Raynor has served as a coinvestigator and consultant on several grants funded by NIH and AHA, in which the primary location of the grants were Rutgers University, Cincinnati Children's Hospital, SUNY Buffalo and Brown University. Her work has also been funded by Weight Watchers, International. She serves as a lecturer for which she receives compensation from the Academy of Nutrition and Dietetics.
